# Boosted stepwise NO photorelease with overall fluorescent reporting in a molecular dyad for antimicrobial photoinactivation

**DOI:** 10.1039/d6ra09028a

**Published:** 2026-10-07

**Authors:** Samantha Sollima, Tassia J. Martins, Cristina Parisi, Francesca Laneri, Juliana Guerra Pinto, Isabelle de Paula Ribeiro Brambilla, Juliana Ferreira-Strixino, Salvatore Sortino

**Affiliations:** a PhotoChemLab, Department of Drug and Health Sciences, University of Catania I-95125 Italy ssortino@unict.it; b Laboratory of Photobiology Applied to Health, Research and Development Institute, University of Vale do Paraíba Urbanova I-2911 Brazil

## Abstract

We have designed and synthesized a molecular dyad in which an *N*-nitroso-nitroaniline-based nitric oxide (NO) photodonor (NOP), capable of releasing two NO molecules through a stepwise mechanism, is covalently linked to an anthracene (An) unit *via* a methylene spacer. The dyad exhibits absorption and emission features that are not merely the sum of its individual components, but rather the result of strong electronic interactions in both the ground and excited states. Excitation of the dyad with blue light significantly enhances the sequential NO photogeneration efficiency compared to the isolated NOP. This effect is likely encouraged by photoinduced electron and energy transfer processes, with An acting as the donor and NOP as the acceptor, providing additional activation pathways to the direct NOP excitation. Furthermore, these intramolecular processes cause a dramatic quenching of the typical anthracene fluorescence, which is fully restored only upon the release of the second NO molecule, thereby enabling the system to act as a fluorescent self-reporter for the overall NO release. Antibacterial tests on Gram-positive methicillin-resistant *Staphylococcus aureus* and Gram-negative *Pseudomonas aeruginosa* demonstrate that photoexcitation of the dyad induces remarkable antibacterial activity against both planktonic bacterial strains and the eradication of *Pseudomonas aeruginosa* biofilms due to the photodynamic effect of NO.

## Abstract 2

Sequential and amplified NO release with fluorescent self-reporting is achieved with photoactivatable molecular dyad exhibiting strong antibacterial actions against Gram positive and Gram negative bacteria.

## Graphical abstract

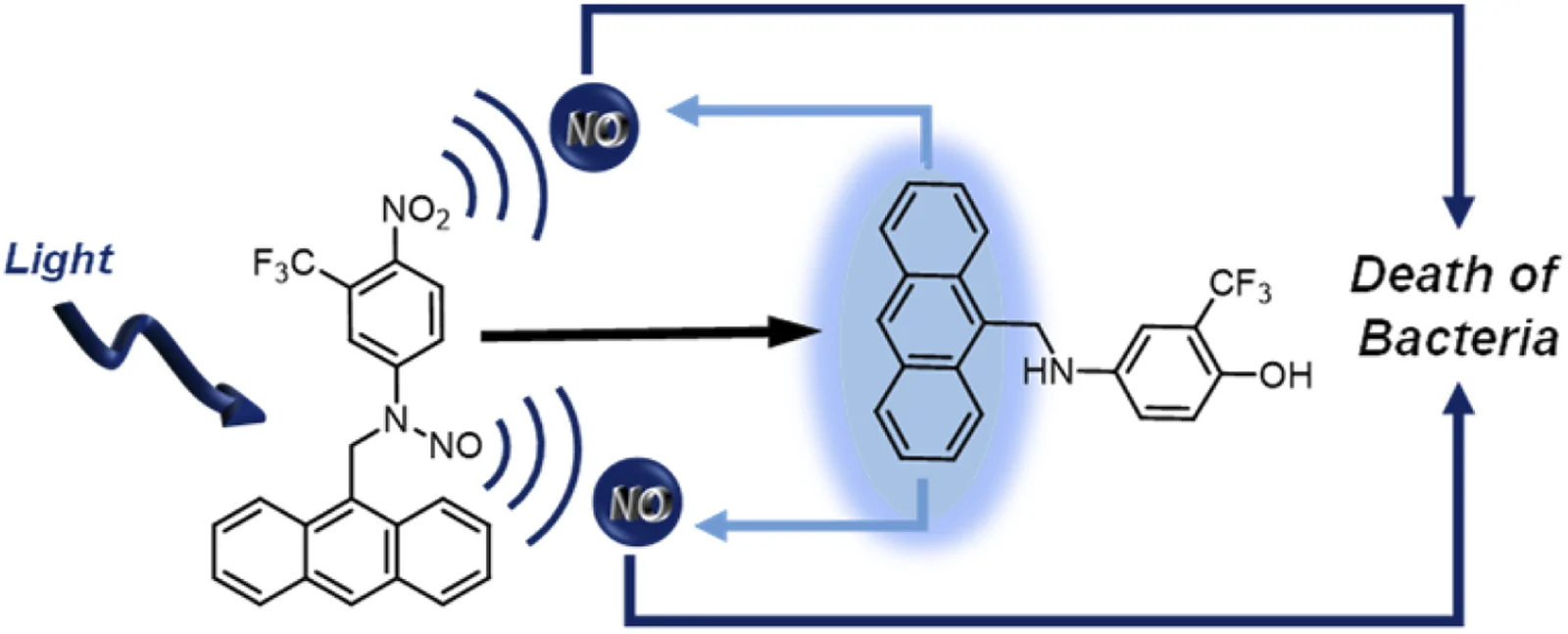

## Introduction

Bacterial infections remain one of the most pressing challenges in modern medicine, largely driven by the rapid global emergence of multidrug-resistant (MDR) pathogens.^1–3^ Conventional antimicrobial therapies, which typically target single essential cellular pathways, frequently induce selective pressure that accelerates resistant mechanisms, including enzymatic degradation, efflux pump activation, and biofilm formation.^4–7^ These important limitations, coupled with the slow pipeline for new clinically approved antibiotics, make the development of novel antibacterial strategies that bypass classic resistance pathways an urgent imperative in healthcare.^8–10^

Nitric oxide (NO) is a low-molecular-weight free radical naturally produced by the innate immune system which, besides its multifaceted role in the bioregulation of vital functions in living bodies,^11,12^ has emerged as a highly promising unconventional antibacterial with a broad spectrum of action^13–15^ due to a combination of unique properties. Unlike traditional and single-target antibiotic drugs, NO does not suffer MDR and exerts its bactericidal and bacteriostatic effects through a complex, multi-targeted mechanism known as nitrosative and oxidative stress.^16^ By indiscriminately damaging bacterial DNA, disrupting cell membrane integrity, and inactivating critical metabolic enzymes, NO minimizes the likelihood of bacteria developing single gene mutational resistance.^17,18^ Furthermore, due to its short half-life (*ca.* 1s)^11,12^ NO offers the advantage of confining its cytotoxic action over short distance (<200 µm), avoiding systemic effects typical of conventional antibiotics.

The intrinsic difficulties in handling NO due to its gaseous nature, have intensively pushed the research over the past two decades towards the development of a large arsenal of NO-releasing precursors.^19–21^ Integrated within tailored molecular, macromolecular, or nanomaterial frameworks, these precursors store NO and typically release it either spontaneously or under appropriate endogenous or exogenous stimuli.^22–27^ Of these, the light-activatable NO precursors,^28–34^ commonly known as NO photodonors (NOPs), are particularly attractive, offering precise spatiotemporal control over NO delivery thanks to the unique advantages of light as powerful and minimally invasive activation tool.^35–37^ This makes NOP very intriguing in photopharmacology, an emerging field that holds high potential to improve the therapeutic outcome while minimizing the off-target side effects.^38,39^ Besides, due to the ephemeral nature of NO and the strict dependence of its biological effects on the concentration and generation site, the development of NOPs with self-reporting functionalities has attracted specific attentions over the last few years.^40,41^ These systems integrate the NOP with a fluorescent unit whose emission is temporarily quenched. Upon activation, the NOP releases a highly fluorescent, stable coproduct simultaneously with NO. This fluorescent product acts as an internal optical self-reporter, enabling the visualization and potential quantification of NO concentration in real time even within cellular environments, without relying on external optical probes,^40,41^ which can yield false positives or negatives.

As part of our longstanding interest focused on NOPs,^30,33,34,40^ we recently reported compound 1, an NOP capable of releasing two NO molecules *via* a stepwise mechanism (Scheme 1A), along with its antibacterial activity.^42^ We also demonstrated that encapsulating 1 within branched cyclodextrin-based polymeric nanocarriers significantly enhances its NO photorelease efficiency as the polymeric network actively acts as H-donor in the radical mediated photorelease pathways.^42^ Based on this work, we present herein a novel strategy to amplify the efficiency of both sequential NO photorelease steps without relying on specific nanocarriers, while simultaneously introducing a fluorescent reporter to monitor the total NO release.

**Scheme 1:**
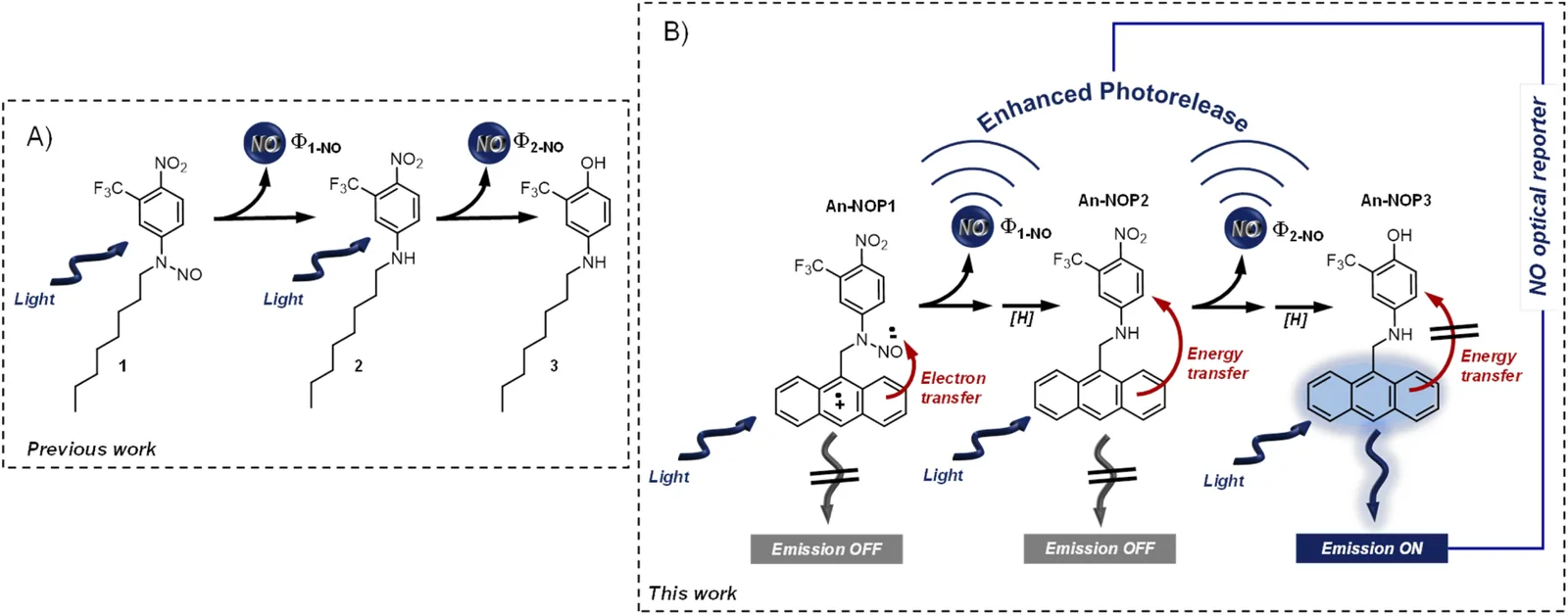
(A) Stepwise NO photorelease from the NOP 1 as reported in ref. 42. (B) The molecular dyad An-NOP1 and its working principle for the enhanced stepwise NO photorelease with overall NO fluorescent reporting.

This was accomplished by designing and synthesizing the molecular dyad An-NOP1 in which the same chromophoric motif of the NOP 1 is covalently linked to an anthracene (An) unit through a methylene spacer, and whose working principle is illustrated in Scheme 1B. Basically, this boosting is driven by photoinduced electron and energy transfer processes, involving the An unit as the donor and NOP moiety as the acceptor, offering additional activation pathways beyond direct NOP excitation (*vide infra*). Furthermore, these intramolecular processes strongly quench the typical An fluorescence, which is restored concomitantly to the release of the second NO molecule. This enables the system to serve as a fluorescent self-reporter for the complete NO release. The dyad was biologically validated through antibacterial assays against planktonic forms and biofilms of Gram-positive methicillin-resistant *Staphylococcus aureus* (MRSA) and Gram-negative *Pseudomonas aeruginosa* (*P. aeruginosa*).

## Results and discussion

An-NOP1 was easily prepared in a two-step synthesis as reported in SI. Fig. 1 shows the absorption spectrum of An-NOP1 and, for comparison, those of the free An and NOP 1, used as suitable model compounds, and their arithmetic sum. Interestingly, the absorption features of the dyad differ from the simple superposition of the absorption of the two isolated components, being characterized by hypochromism of the bands associated with both chromophoric components and by a shift toward longer wavelengths, which is more clearly observed in the case of the typical structured absorption band of the An moiety. Due to the presence of the methylene spacer insulator, the absorption of An-NOP1 cannot be ascribed simply to changes in the molar extinction coefficient after the covalent linking of the two chromophoric units. Instead, it accounts for a strong electronic interaction between An and the NOP moieties in the ground state. This was confirmed by the absence of hypochromism and red shift in the case of the physical mixture of An and the NOP 1 under otherwise experimental conditions, whose absorption spectrum was basically the same as their arithmetic sum already reported in Fig. 1.

**Fig. 1:**
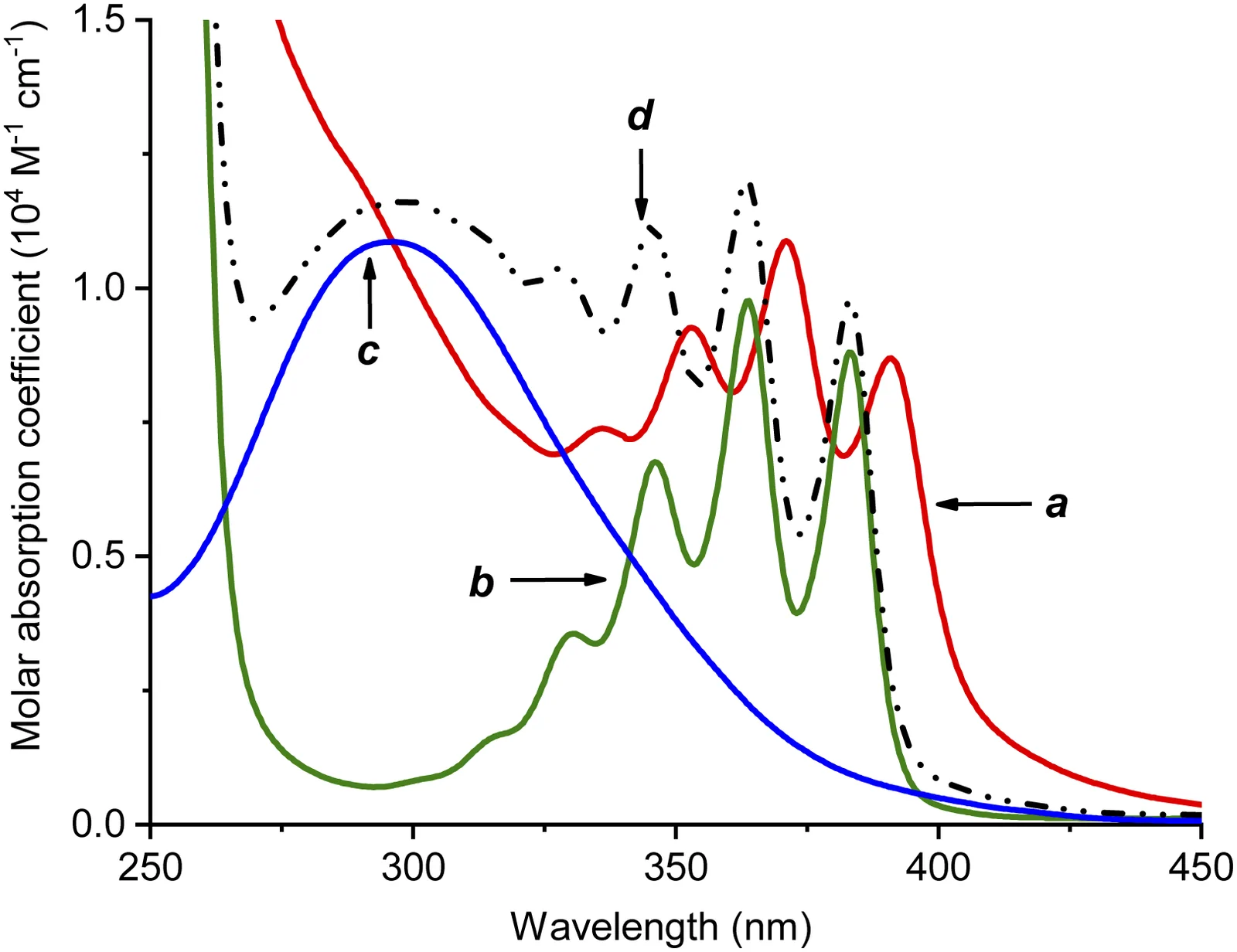
Absorption spectra of An-NOP1 (a), An (b), NOP 1 (c) and the arithmetic sum of b and c (d); CH_3_OH : H_2_O 75 : 25 (v/v); 25 °C.

The dyad was very stable in the dark (Fig. S9) but responsive to light excitation. The photolysis profile is clearly biphasic with a first, faster step characterized by a bleaching of the absorption below 320 nm accompanied by a remarkable increase of the band at 390 nm (Fig. 2A), and a second, slower photolytic step characterized by the bleaching of this 390 nm absorption (Fig. 2B). Both steps show precise isosbestic points, which are indicative of very clean photochemical processes. Fig. 2C better shows the two distinct photolysis pathways occurring at different rate. The absorption spectrum observed after the first step of photolysis matches well that of the non-nitrosated dyad An-NOP2 (Fig. S10), characterized by the restoring of the push pull character of the nitroaniline moiety. Formation of this compound as the sole stable photoproduct in the initial part of the photolysis was unambiguously confirmed by HPLC analysis carried out with the authentic An-NOP2 sample and the irradiated mixture (traces a–e Fig. 2D), accounting for the loss of NO from the nitroso moiety and H transfer from solvent to the anilinyl radical intermediate, typical for *N*-nitrosoaniline derivatives.^43–48^

**Fig. 2:**
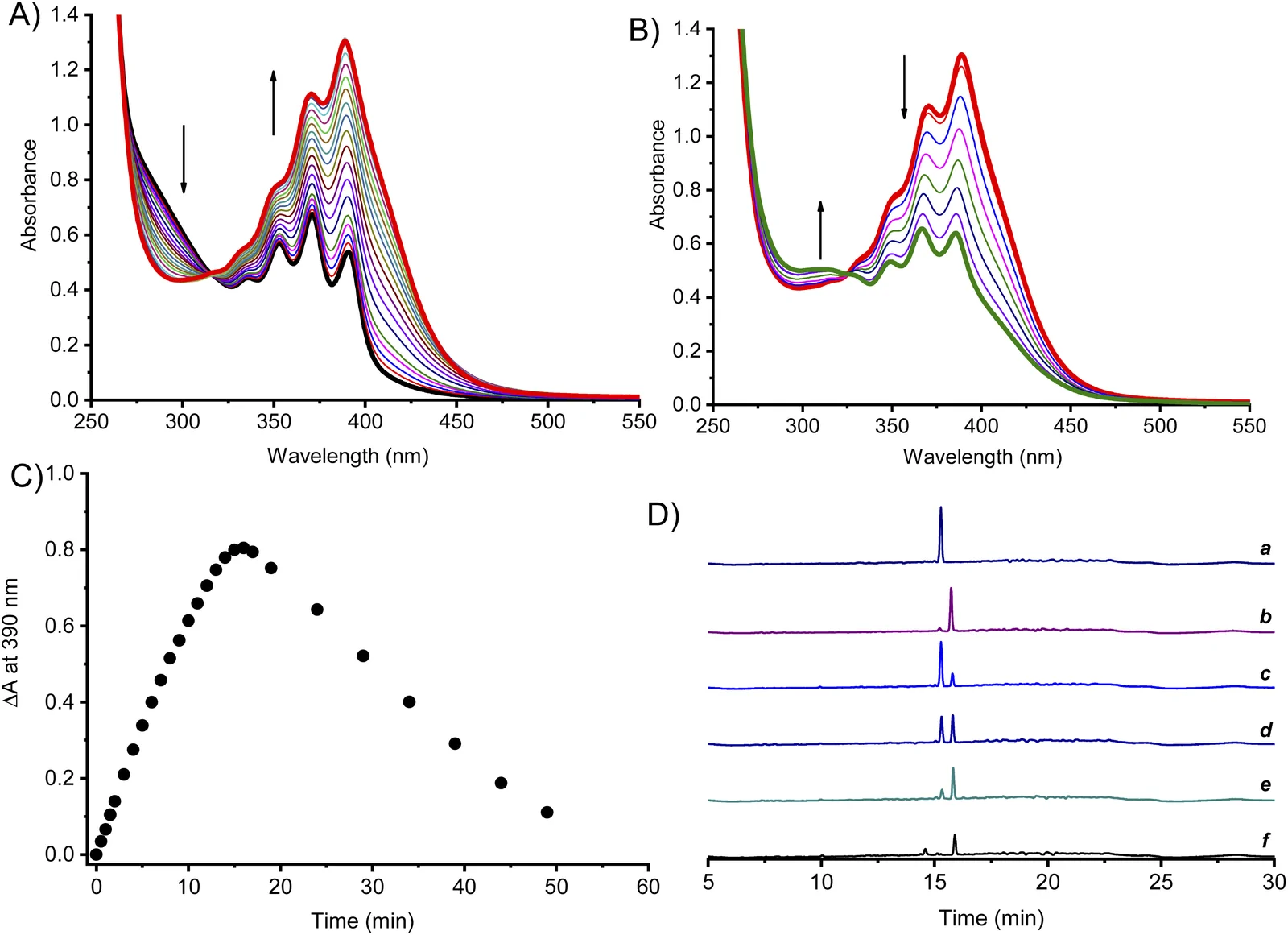
Absorption spectral changes observed upon light exposure of an air-equilibrated solution of An-NOP1 (60 µM) at *λ*_exc_ = 405 nm (*ca*. 100 mW cm^−2^) at different irradiation times from 0 to 18 min (A) and from 18 min to 49 min (B). The arrows indicate the course of the spectral profile with the illumination time. CH_3_OH : H_2_O 75 : 25 (v/v); *T* = 25 °C. (C) Absorbance changes at 390 nm related to the photolysis profiles reported in (A) and (B). (D) HPLC traces related to solutions of An-NOP1 (a), An-NOP2 (b), An-NOP1 after 4 min (c), 10 min (d), 16 min (e) and 34 min (f) of irradiation at *λ*_exc_ = 405 nm (*ca*. 100 mW cm^−2^) recorded at *λ* = 320 nm.

Regarding the second photolytic step, HPLC analysis confirmed the presence of only one photoproduct (trace f in Fig. 2D), consistent with the isosbestic point observed (see Fig. 2B). The spectral evolution characterizing this second photolytic step closely resembles that typically observed for the irradiation of several derivatives of the 4-nitro-3-(trifluoromethyl)aniline chromophoric scaffold extensively used in our group.^49–51^ NO photorelease from this unit leads to phenol derivatives absorbing in the UV region and responsible for the photobleaching observed.^49–51^ On these basis, An-NOP3 can be reasonably and tentatively proposed as the sole photoproduct formed in the second photolysis step.

NO generation was confirmed by the typical fluorimetric assay based on the use of 2,3-diaminonaphthalene (DAN) as a probe, one of the most sensitive and selective optical methods for NO detection as nitrite generated after spontaneous NO oxidation.^52^ We performed this test on the irradiated mixture almost at the end of the first and the second step of photolysis and, for comparison, on the non-irradiated sample. Fig. 3 shows the appearance of the diagnostic fluorescence emission and excitation of the 2,3-diaminonaphthotriazole (DANT) formed after reaction of DAN with nitrite, only in the irradiated sample. The satisfactory ratio of the emission intensity at the two irradiation times provides convincing evidence for the multiple photorelease of NO. Interestingly, the quantum yields related to the two sequential processes were calculated to be *Φ*_1-NO_ = (4 ± 0.3) × 10^−2^ and *Φ*_2-NO_ = (1.4 ± 0.2) × 10^−3^, respectively. These values are *ca.* 10 and 2-fold larger than those previously observed for the isolated NOP 1 under similar experimental conditions.^42^ From these data, it appears that an additional photoactivation pathways beyond direct NOP excitation may be involved in the NO release. The red shift of the absorption spectrum of the dyad An-NOP1 compared with the isolated components (see Fig. 1) suggests that, beside the *N*-nitroso-nitroaniline also the An chromophore is able to absorb the excitation light. In this view, we believe that a probable explanation for this enhancing effect could involve a direct participation of the An moiety in intramolecular photoinduced processes. Specifically, as far as the first step is concerned, a photoinduced electron transfer between the An, as electron donor, and the *N*-nitroso-moiety, as electron acceptor, of the An-NOP1 dyad leading to the formation of NO-centered radical anion can be reasonably responsible for the NO release and, after back electron transfer and H transfer from the solvent, for the formation of An-NOP2 as stable photoproduct (Scheme 1B). This hypothesis is supported by the following:

**Fig. 3:**
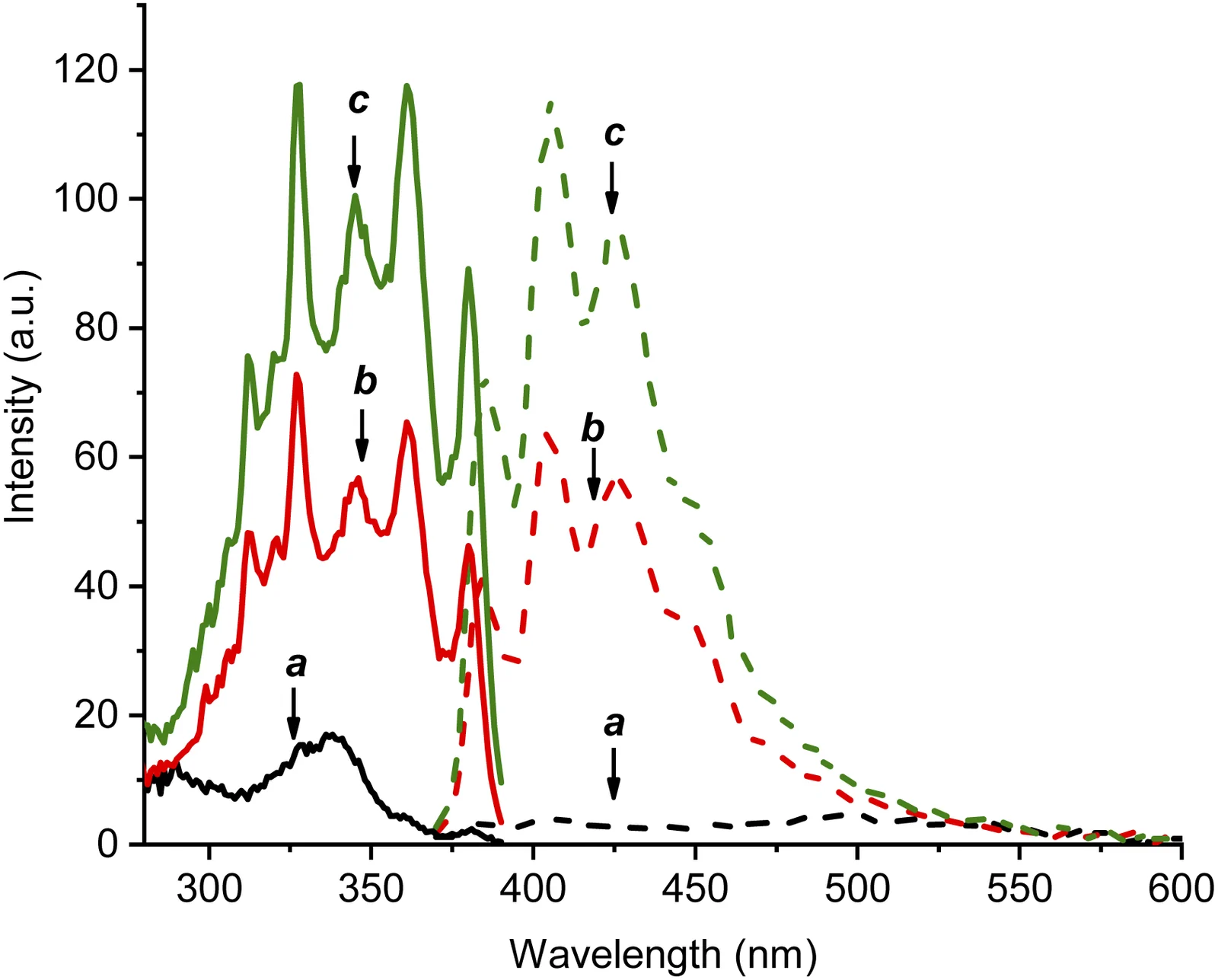
Fluorescence emission (dotted lines) and excitation (solid lines) spectra obtained after fluorimetric assay of An-NOP1 solutions kept in the dark (a) and irradiated 18 min (b) and 49 min (c) in CH_3_OH : H_2_O 75 : 25 (v/v) under air-equilibrated conditions. The *λ*_em_ for excitation spectra was 410 nm whereas the *λ*_exc_ for the emission spectra was 360 nm. *T* = 25 °C.

(i) The negative free energy value for the photoinduced electron transfer processes, Δ*G*∼ − 1.1 eV, estimated by the Rehm–Weller eqn (1):^53^

1 Δ*G* = e[*E*_ox_ − *E*_red_] − *E*_0,0_

where *E*_ox_ is the half-wave potential for one-electron oxidation of the electron donor unit, *ca.* 1.5 eV *vs.* NHE,^54^*E*_red_ is the half-wave potential for one-electron reduction of the electron acceptor unit, *ca.* −0.4 eV *vs.* NHE,^55^ and *E*_0,0_ is the average energy of the lowest excited singlet states of An, *ca*. 3 eV;^56^

(ii) The complete suppression of the typical fluorescence emission of An (Fig. S11) as a result of this highly competitive deactivation mechanism;

(iii) The fact that radical anions of nitroso-derivatives possess a lower dissociation energy of the N–NO bond than their neutral form, encouraging fast NO detachment;^57^

(iv) The occurrence of this photoinduced mechanism recently observed by us and others with similar nitroso-derivatives combined with various light harvesting antennae.^55,58,59^

As far as the second step is concerned, we believe that, besides the direct excitation of the nitroaniline chromophore of the dyad An-NOP2, a photoinduced energy transfer between the An moiety and the nitroaniline chromophore can further encourage the NO release from this latter, as illustrated in Scheme 1B. This hypothesis is supported by the following:

(i) The considerable overlap between the fluorescence emission spectrum of the An (energy donor) and the absorption spectrum of the nitroaniline (energy acceptor) components of the dyad (Fig. S12), indispensable prerequisite for an energy transfer occurring through a FRET mechanism;^60^

(ii) The close proximity of the donor and the acceptor;^60^

(iii) The complete suppression of the typical fluorescence emission of the An in the dyad An-NOP2 (Fig. S11).

(iv) The occurrence of a similar FRET mechanism that we already demonstrated with a similar dyad separated by a longer alkyl spacer.^61,62^

Fig. 4 shows the evolution of the fluorescence emission spectra corresponding to the absorption changes reported in Fig. 2A and B and covering the entire time window of the photolysis. It can be noted a dramatic revival of the characteristic emission arising from the anthracene fluorophore upon light excitation. The inset of Fig. 4 and its comparison with Fig. 2C, indicates the relevant increase of fluorescence starting after *ca.* 18 min of irradiation that represents the beginning of the second step of photolysis. This behavior is in excellent agreement with the negligible emission of An-NOP1 and An-NOP2 (see Fig. S11) and in accordance with the formation of the phenol derivative An-NOP3 as stable photoproduct after the release of the second NO molecule (see Scheme 1B). In fact, in contrast to the nitroaniline appendage, the phenol unit is not an energy acceptor towards An since its absorption falls in the UV region. This does not allow the spectral overlap with An emission, making the FRET process thermodynamically not feasible and thus restoring the emission of the An unit. As a consequence, as illustrated in Scheme 1B, An-NOP3 can act as an optical self-reporter to monitor the overall amount of NO photoreleased through the stepwise process.

**Fig. 4:**
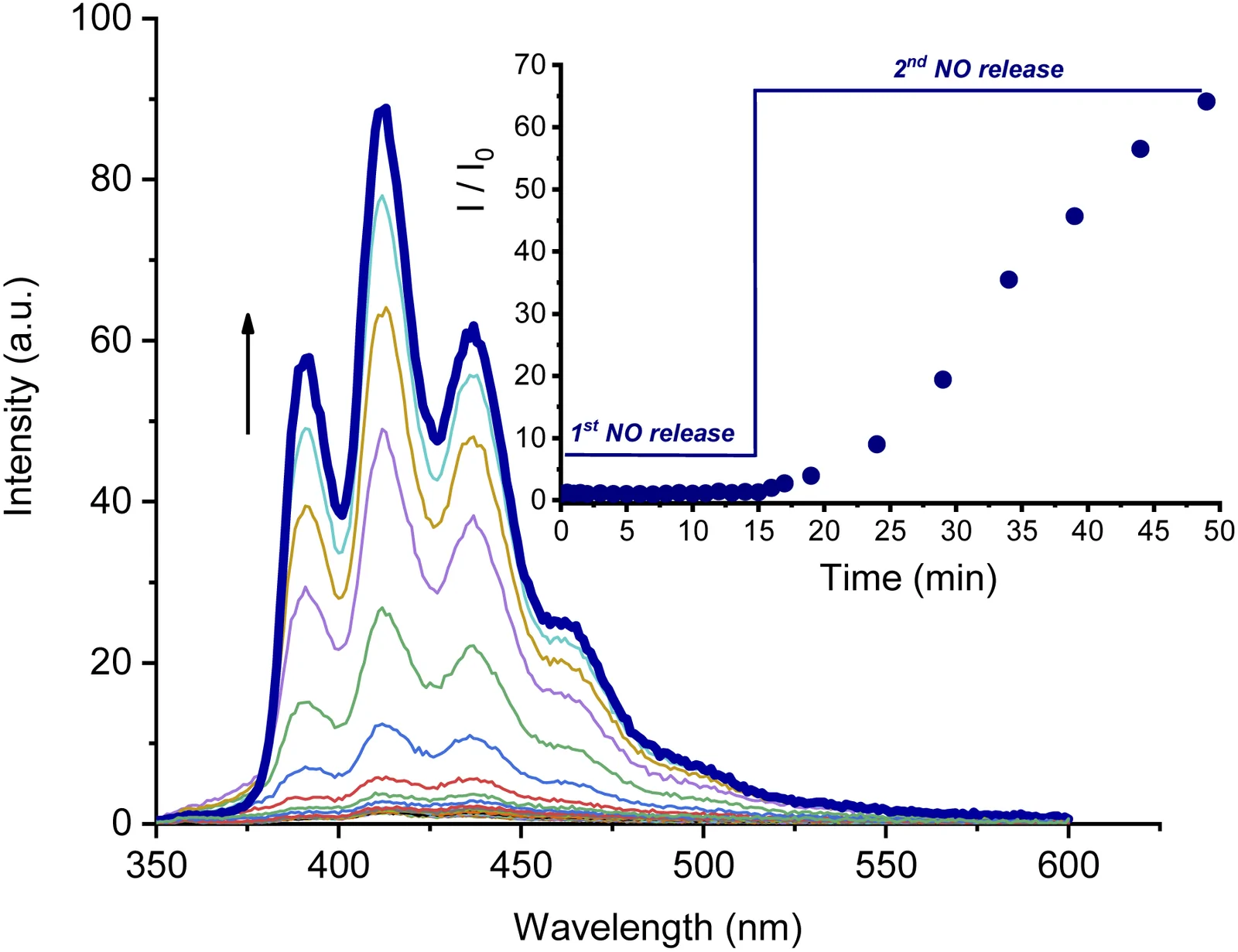
Evolution of the fluorescence emission spectra corresponding to the sample of Fig. 2A and B (from 0 to 49 min of irradiation) and recorded at *λ*_exc_ = 325 nm. The arrow indicates the course of the spectral profile with the illumination time. The inset shows the dependence of the *I*/*I*_0_ ratio (*I* and *I*_0_ represent the fluorescence intensities after and before each step of irradiation, respectively) as a function of the irradiation time. *T* = 25 °C.

Antibacterial activity of the dyad An-NOP1 was tested against planktonic forms and biofilms of methicillin-resistant *Staphylococcus aureus* (MRSA) and *P. aeruginosa*, which are specimens of Gram-positive and Gram-negative bacteria responsible for the higher rate of disease due to the high antibiotic resistance pattern towards conventional antibiotics. Fig. 5A reports the results obtained for the bacterial cultures either kept in the dark or irradiated with a blue LED. Based on the light fluence used, both NO photorelease steps are expected to occur. The planktonic assays demonstrated that the dyad exhibited strong antibacterial activity against both bacterial strains, reducing viable bacterial counts below the detection limit (LOD) (1 × 10^6^ CFU mL^−1^, corresponding to 6.00 log CFU mL^−1^) at both tested concentrations upon irradiation. In contrast, no bacterial inactivation has been observed under dark conditions, suggesting the active photodynamic role of NO in the bacterial growth inhibition. Note that, as reported in our recent work, the free NOP 1 (Scheme 1A) did not exhibit any photodynamic action unless delivered by polymeric nanocarriers, accounting for a key role of the An component in the observed photoinduced antibacterial action.

**Fig. 5:**
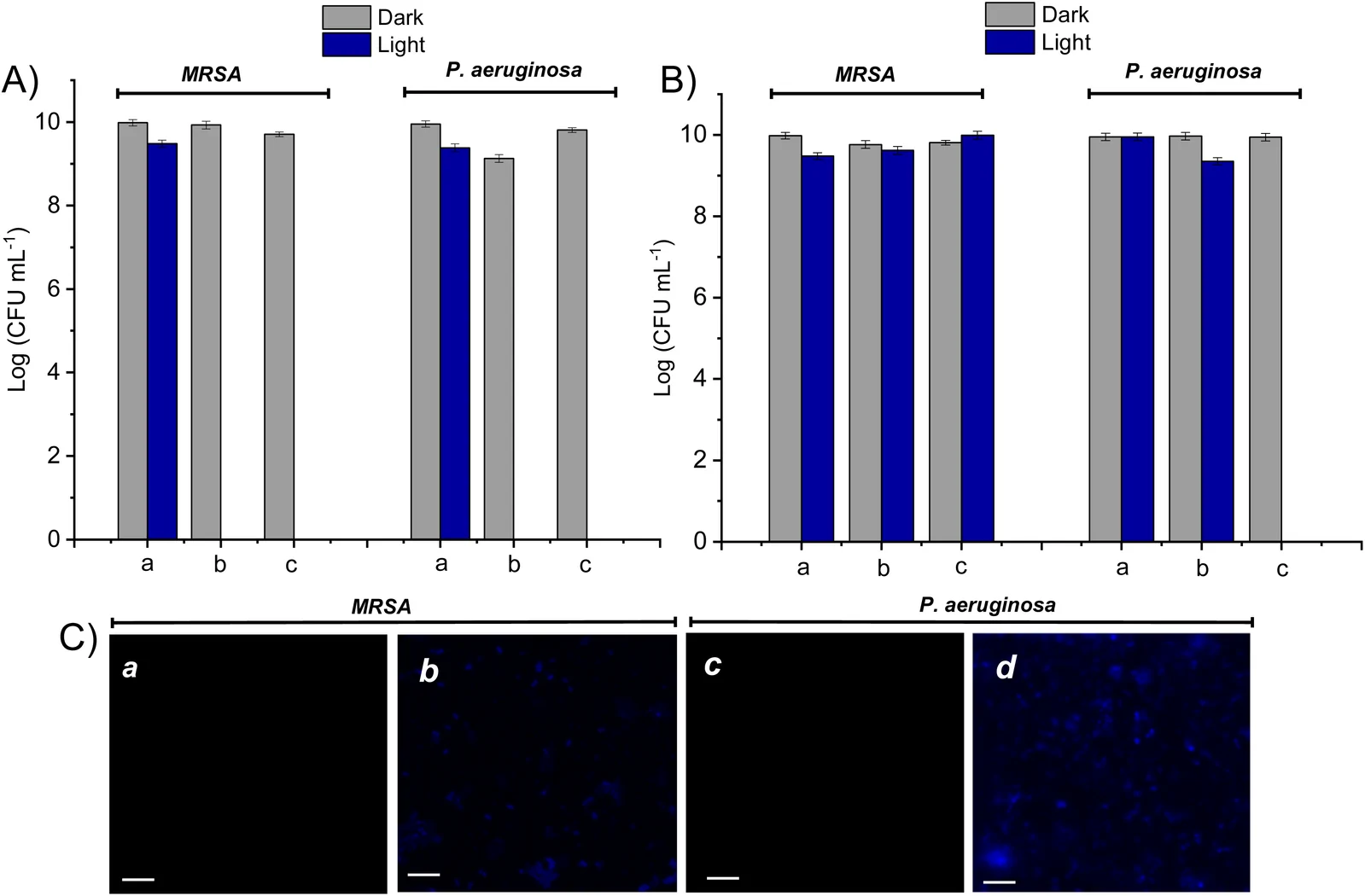
Planktonic (A) and biofilm bacteria assay (B) for MRSA and *P. aeurginosa* incubated with 0 µM (a), 25 µM (b) and 50 µM (c) An-NOP1 and either kept in the dark or irradiated for 40 min with a blue LED (*ca*. 45 mW cm^−2^). Note: the log(CFU mL^−1^) in the irradiated samples b and c in (A) and sample c for *P. aeruginosa* in (B) cannot be distinguished from the *x* axis. The LOD of the colony-counting assay was 1 × 10^6^ CFU mL^−1^ (6.00 log CFU mL^−1^). Samples yielding no detectable colonies are indicated as below the LOD and should not be interpreted as having zero viable bacteria. (C) Confocal microscopy images of MRSA and *P. aeurginosa* biofilm bacteria incubated with An-NOP1 (50 µM) in the dark and taken before (panels a and c) and after (panels b and d) 40 min of irradiation with a blue LED. Note: no appearance of blue fluorescence was observed upon cell irradiation in the absence of An-NOP1. Bar = 5 µm.

Since planktonic cells have a different mechanism and lack the protective barriers characteristic of biofilms, the antibiofilm activity was also evaluated. Fig. 5B shows that the dyad did not significantly reduce bacterial viability at the tested concentrations, either in the dark or under irradiation. In contrast, *P. aeruginosa* biofilms were susceptible to An-NOP1 at 50 µM exclusively upon irradiation, with viable bacterial counts below the LOD, corresponding to a minimum reduction of 2.26 log relative to the irradiated control. Interesting insights into this different behavior were obtained by confocal microscopy analysis exploiting the photogeneration of the NO fluorescent reporter discussed above. Fig. 5C shows representative confocal microscopy images taken before and after irradiation of the two types of biofilms incubated with An-NOP1. It can be seen the appearance of the typical blue emission of the fluorescent reporter much more intense in the case of *P. aeruginosa* rather than MRSA. This result provides a clear-cut indication not only for the preservation of the photochemical behavior of the dyad in the biological environment, which is not a trivial result, but also for its more effective internalization in the case of *P. aeruginosa* which well accounts for the observed antibacterial action.

## Conclusions

In summary, we have reported a photoactivatable molecular dyad releasing two NO molecules upon blue light irradiation through a stepwise process significantly boosted compared to the isolated NOP by thermodynamically favored intramolecular photoinduced electron and energy transfer processes between the two chromophoric components. The dyad is non-emissive but the fluorescence is remarkably restored upon the release of the second NO molecule, permitting the real-time optical monitoring of the overall NO release even in a biological environment. In contrast with the individual NOP component, the dyad exhibits a powerful antibacterial photodynamic action against both planktonic MRSA and *P. aeruginosa* and is able to completely eradicate the *P. aeruginosa* biofilms due to its more effective internalization and the photodelivery of NO therein.

## Experimental

### Chemicals

4-Nitro-3-(trifluoromethyl) aniline, 9-(chloromethyl)anthracene, and sodium nitrite were purchased from Sigma-Aldrich and used as received. Solvents employed in the synthetic procedures were of high purity grade, while solvents used for spectrophotometric studies were of spectrophotometric grade.

### Instrumentation

^1^H, ^13^C NMR, gCOSY and HSQC spectra were recorded on a Varian UNITY Inova at 500 MHz. Chemical shifts (*δ*) are given in parts per million (ppm) and the coupling constants (*J*) are given in Hz. The following abbreviations are used to designate peak multiplicity: s = singlet, bs = broad singlet, d = doublet, dd = doublet of doublets, t = triplet, q = quartet, quint = quintuplet, m = multiplet.

Flash column chromatography was performed on silica gel (Merck Kieselgel 60, 230–400 mesh ASTM). The reactions progress was monitored by thin layer chromatography (TLC) on 5 × 20 cm plates with a layer thickness of 0.2 mm.

UV-vis absorption spectra and fluorescence emission spectra were recorded with a JascoV-560 spectrophotometer and a Spex Fluorolog-2 (mod. F111) spectrofluorimeter, respectively in air-equilibrated solutions, using either quartz cells with a path length of 1 cm.

The reverse-phase HPLC analyses were performed using a Jasco LC-4000 series system, consisting of a non-thermostated autosampler (AS-4050), a thermostated column compartment (CO-4061), a quaternary pump (PU-4180), and a diode array detector (MD-4010).

The analytical column was a SUPELCOSIL C-18 (15 cm × 4.6 mm × 5 µm). The mobile phases A and B consisted of water and acetonitrile, respectively. A gradient elution was performed starting at 40% B, linearly increased to 90% B over 15 minutes, held for 5 minutes, then returned to the initial conditions within 5 minutes and held for a further 5 minutes. The flow rate was 1.0 mL min^−1^, and elution was monitored at 320 nm.

Photolysis experiments were performed by irradiating the samples in solution in a thermostated quartz cell (1 cm pathlength, 3 mL capacity) under gentle stirring, using a blue laser (*λ*_exc_ = 405 nm) with an irradiance on the samples of *ca.* 100 mW cm^−2^.

### Photolysis quantum yields

Quantum yields for the photolytic steps (*Φ*) were determined at *λ*_exc_ = 405 nm within the 20% transformation, determined by HPLC, through eqn (2):

2 *Φ* = [*X*] × *V*/*t* × (1 − 10^−*A*^) × *I*

where, [*X*] is the concentration of the phototransformed An-NOP1 or An-NOP2, *V* is the volume of the irradiated sample, *t* is the irradiation time, *A* is the absorbance of the sample at the excitation wavelength and *I* the intensity of the excitation light source.

### NO detection

NO was measured by means of the well-known, highly sensitive (detection limit on the order of the picomoles) fluorimetric bioassay of Misko *et al.*,^52^ based on the ring closure of the DAN with nitrite to form the highly fluorescent product DANT. Aliquots of 2 mL of solutions of An-NOP1 were kept in the dark or irradiated at different times. Afterwards, the samples were treated with DAN according with the standard protocol.^52^

### Planktonic assays

Bacterial strains of Gram-positive MRSA and Gram-negative *P. aeruginosa* were replicated in a Falcon tube containing BHI broth, which was incubated for 24 hours in an incubator at 37 °C. After subculturing, the cultures were centrifuged at 3500 rpm for 15 min, the supernatant was discarded, and the bacterial suspensions were standardized to 0.5 on the McFarland scale. Following standardization, 2000 µL of the bacterial suspension was transferred to Falcon tubes according to the experimental groups. The compounds were solubilized in PBS containing 0.5% (v/v) DMSO. The tubes were then centrifuged, and the resulting pellets were resuspended in 800 µL of the tested compounds. For the control, phosphate-buffered saline (PBS) was used instead of the compounds. Aliquots of 100 µL from each suspension were dispensed in triplicate into a 96-well plate. The plate for the treated groups was then irradiated with a 420 nm LED (44 mW cm^−2^). After treatment, serial dilutions were performed, and the samples were plated onto BHI agar plates using the drop plate technique. The plates were incubated at 37 °C for 24 h, after which colony-forming units (CFU) were counted.

The detection limit of the colony-counting assay was calculated based on a plated volume of 10 µL at a dilution of 10^−4^, considering one colony as the minimum detectable count. Under these conditions, the detection limit was 1 × 10^6^ CFU mL^−1^ (6.00 log CFU mL^−1^). Samples yielding no detectable colonies were reported as having viable bacterial counts below the detection limit.

### Biofilm formation and assay

Bacterial strains of MRSA and *P. aeruginosa* were subcultured in brain heart infusion (BHI) broth prior to biofilm formation. Biofilms were developed in sterile 96-well plates. After subculturing, the bacterial cultures were centrifuged at 3500 rpm for 15 min, and the supernatant was discarded. This procedure was repeated, and the bacterial suspensions were subsequently standardized to 0.5 on the McFarland scale. Afterward, 22.5 µL of the bacterial suspension was added to 200 µL of BHI broth in each well. The plates were incubated at 37 °C for 24 h to allow biofilm formation. After incubation, the culture medium was carefully removed, and 100 µL of each tested compound (0.5% DMSO in PBS) was added to the respective wells. For the control, phosphate-buffered saline (PBS) was added instead of compounds. The plates corresponding to the treated groups were then irradiated with a 420 nm LED (44 mW cm^−2^). After irradiation, biofilms from both irradiated and non-irradiated plates were detached by mechanical disruption, followed by serial dilution in sterile diluent. Aliquots from each dilution were plated onto BHI agar plates using the drop plate technique. The agar plates were incubated at 37 °C for 24 h, and colony-forming units (CFU) were counted.

### Confocal microscopy

After bacterial standardization, a coverslip was placed in each well of a 24-well plate according to the experimental groups. Subsequently, 225 µL of the bacterial suspension and 2000 µL of BHI broth were added onto each coverslip, and the plate was incubated at 37 °C for 24 h. After incubation, the medium was removed, and the wells were gently washed with phosphate-buffered saline (PBS). Fresh BHI broth was then added, followed by an additional incubation period of 24 h at 37 °C. After this period, the medium was removed, the tested compounds were added, and the plate was irradiated with a 450 ± 20 nm LED (44 mW cm^−2^). Following irradiation, the compounds were removed, and the biofilms were fixed with 4% paraformaldehyde for 15 min. The fixative was then removed, and the coverslips were mounted onto glass slides using a drop of Entellan. Confocal images were acquired using laser excitation at 405 nm on a Zeiss LSM 700 confocal microscope.

## Author contributions

S. S. carried out most of the spectroscopic and photochemical experiments; T. J. M. carried out the synthesis of the compounds and some photochemical experiments; C. P. and F. L. supervisioned the spectroscopic and photochemical experiments, the synthesis and structural characterization procedures; J. G. P. and I. d. P. R. P. performed the antibacterial tests and the fluorescence microscopy analysis; J. F. S. supervisioned the biological experiments; S. S. devised the work, designed the experiments and wrote the paper.

## Conflicts of interest

We have no conflict of interest to declare.

## Supplementary Material

RA-OLF-D6RA09028A-s001

## Data Availability

The data supporting this article have been included as part of the supplementary information (SI). Supplementary information: NMR Spectra, absorption and emission spectra not reported in the main text. Additional raw data supporting the findings of this study are available from the corresponding author upon request. See DOI: https://doi.org/10.1039/d6ra09028a.
