# Supplementary material for: Boosted stepwise NO photorelease with overall fluorescent reporting in a molecular dyad for antimicrobial photoinactivation
Source: RSC Adv. 2026 Oct 7;16(46):60622–30. doi: 10.1039/d6ra09028a (PMC13643699; doi:10.1039/d6ra09028a)
Supplement: Supplementary file 1 [file RA-016-D6RA09028A-s001.pdf]

Supplementary Information (SI) for RSC Advances.
This journal is © The Royal Society of Chemistry

Electronic Supplementary Information

Boosted Stepwise NO Photorelease with Overall Fluorescent Reporting in a Molecular Dyad for Antimicrobial Photoinactivation

Samantha Sollima,†[a] Tassia J. Martins,†[a] Cristina Parisi,[a] Francesca Laneri,[a] Juliana Guerra Pinto,[b] Isabelle de Paula Ribeiro Brambilla,[b] Juliana Ferreira-Strixino,[b] and Salvatore Sortino*[a]

[a]*PhotoChemLab, Department of Drug and Health Sciences, University of Catania, I-95125 Catania, Italy*
[b]*Laboratory of Photobiology Applied to Health, Research and Development Institute, University of Vale do Paraíba, Urbanova I-2911, Brazil*

† Contributed equally
* Email: ssortino@unict.it

Table of Contents

Synthesis....S2
Scheme 1 ....S2
Figure S1 ....S3
Figure S2 ....S3
Figure S3 ....S4
Figure S4 ....S4
Figure S5 ....S5
Figure S6 ....S5
Figure S7 ....S6
Figure S8 ....S6
Figure S9 ....S7
Figure S10 ....S7
Figure S11 ....S8
Figure S12 ....S8

Synthesis

An-NOP1 was easily prepared in a two-step synthesis as reported in Scheme S1 and described in the following. All operations were carried out under a low-intensity level of visible light.

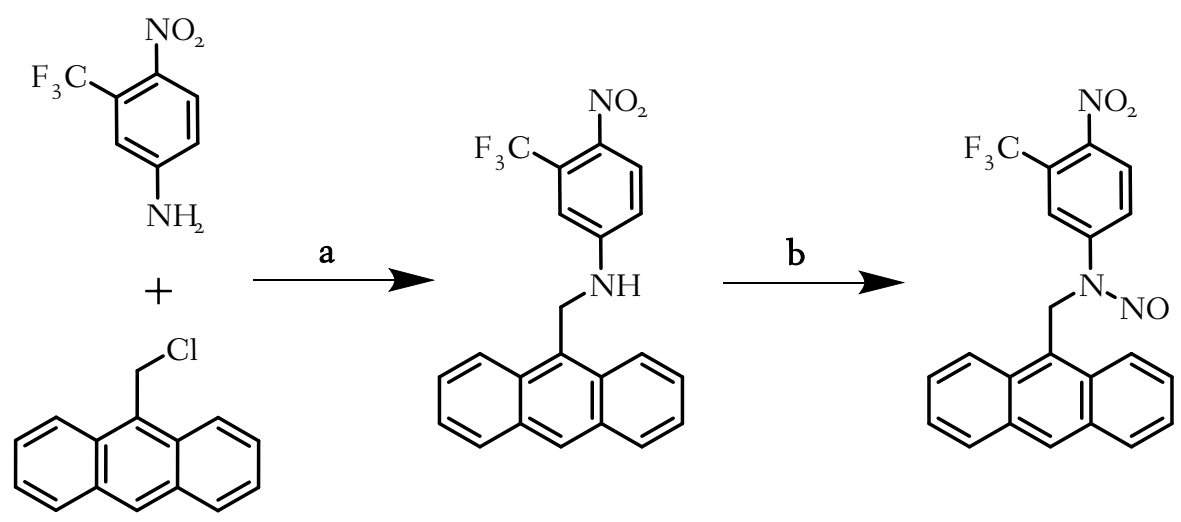

An-NOP2 **An-NOP1**

Scheme S1. a) K_2CO_3, KI, CH_3CN:toluene, 1:1 (v/v) reflux overnight. **b) An-NOP2**, $NaNO_2$, THF:CH_3COOH, 1:1 (v/v) 12 h, 0°C □ r.t.

Synthesis of N-(anthracen-9-ylmethyl)-4-nitro-3-(trifluoromethyl)aniline anthracene-nitroaniline (An-NOP2)

KI (229 mg; 1.38 mmol) and K_2CO_3 (588 mg; 4.25 mmol) were solubilized in a mixture of acetonitrile and toluene 1:1 (v/v, 150 mL), and the solution was heated to reflux (75 °C). 4-nitro-3-(trifluoromethyl)aniline (533 mg, 2.59 mmol) was then added, and the mixture was kept under reflux for 15 minutes. After that, 9-(chloromethyl)anthracene (589 mg; 2.6 mmol) was added, and the reaction was stirred under reflux overnight. After completion, the solvent was evaporated under reduced pressure and the crude purified by flash chromatography eluting with Cyclohexane/Ethyl Acetate (Cy/ EtOAc, 90/1 v/v) yielding **An-NOP2** as a yellow solid (290 mg, 28.2%). The product was characterized by NMR ^{1}H, ^{13}C , gCOSY and HSQC. ^{1}H NMR (500 MHz, CD_2Cl_2) δ 8.57 (s, 1H), 8.21 (d, 2H), 8.13-8.09 (m, 3H), 7.60-7.52 (m, 4H), 7.08 (d, 1H), 6.95-6.93 (dd, 1H), 5.29 (d, 2H), 4.83 (s, 1H). ^{13}C NMR (126 MHz, CD_2Cl_2) δ 152.11, 131.94, 130.85, 129.73, 129.63, 129.25, 127.44, 127.22, 125.77, 123.84, 113.33, 111.59, 40.79. NMR spectra are shown in Figures S1, S2, S3, S4.

Synthesis of N-(anthracen-9-ylmethyl)-N-(4-nitro-3-(trifluoromethyl)phenyl) nitrosamine (An-NOP1)

The synthesis of **An-NOP1** was performed using a methodology well established in our laboratory (2). **An-NOP2** (90 mg, 0.43 mmol) was solubilized in a mixture of THF and acetic acid 1:1 (v/v, 8 mL) and the solution was cooled down with an ice bath. After, sodium nitrite (241 mg, 3.5 mmol) was added to the solution and kept under stirring at 0 ºC for one hour and at room temperature overnight. The reaction mixture was diluted in dichloromethane (DCM), washed with saturated sodium bicarbonate solution (3 × 20 mL), water (3 × 20 mL), dried over Na_2SO_4 and concentrated to dryness, purified by flash chromatography eluting with Cyclohexane/Ethyl Acetate (Cy/EtOAc, 70/30 v/v) yielding **An-NOP1** as a yellow solid (78 mg, 42.7%). The product was characterized by NMR ^{1}H, ^{13}C, gCOSY and HSQC. ^{1}H NMR (500 MHz, CD_2Cl_2) δ 8.46 (s, 1H), 8.03-8.01 (d, 2H), 7.89-7.87 (d, 2H), 7.61 (m, 2H), 7.55 (d, 2H), 7.50 (m, 3H), 6.23 (s, 2H). ^{13}C NMR (126 MHz, CD_2Cl_2) δ 143.78, 134.53, 131.58, 130.99, 130.17, 130.12, 129.71, 129.48, 128.22, 126.36, 126.13, 125.71, 123.92, 122.88, 122.61, 121.75, 40.28. NMR spectra are shown in Figures S5, S6, S7, S8.

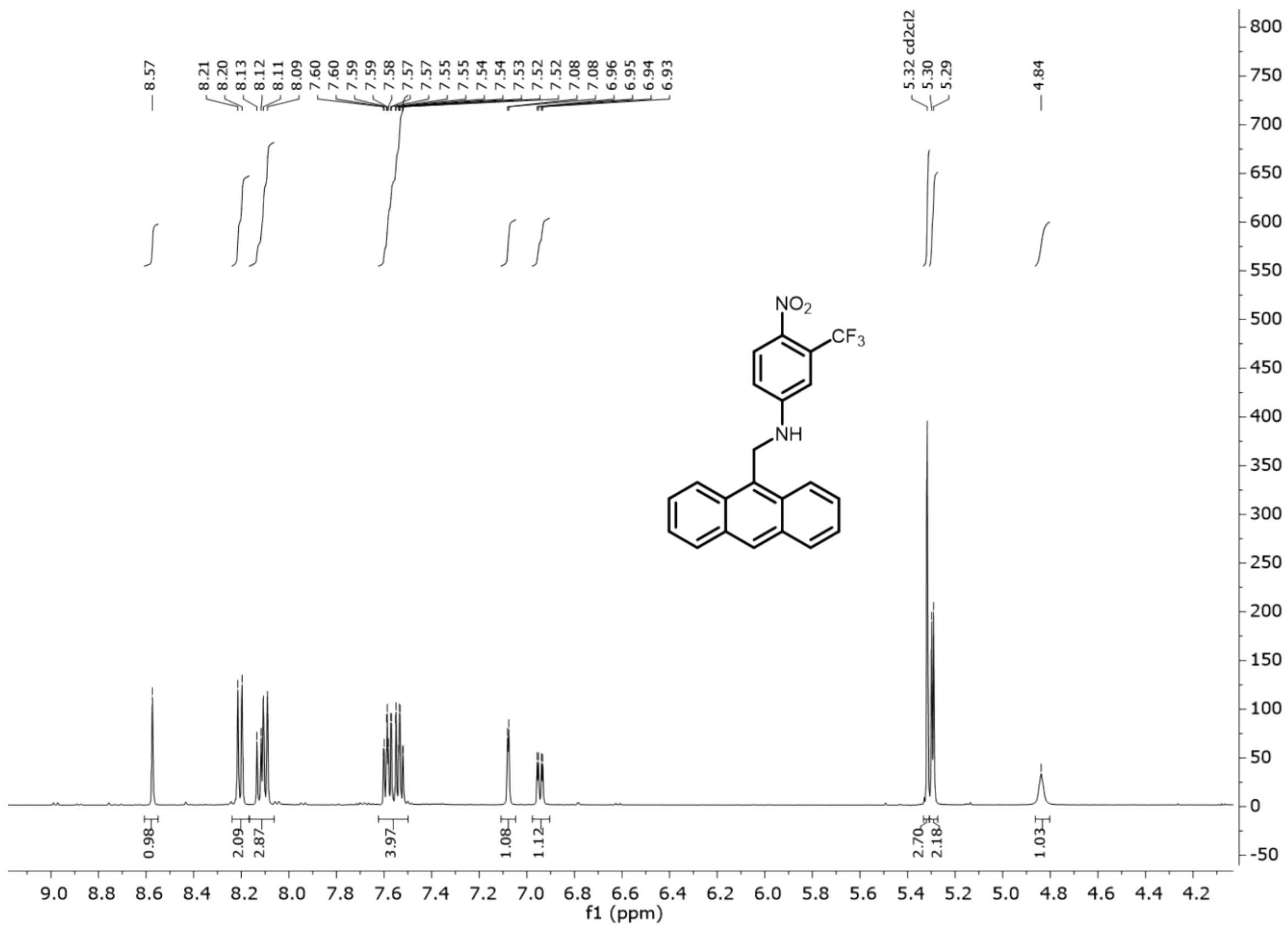

Fig. S1 ^{1}H-NMR spectrum of **An-NOP2** in CD_2Cl_2.

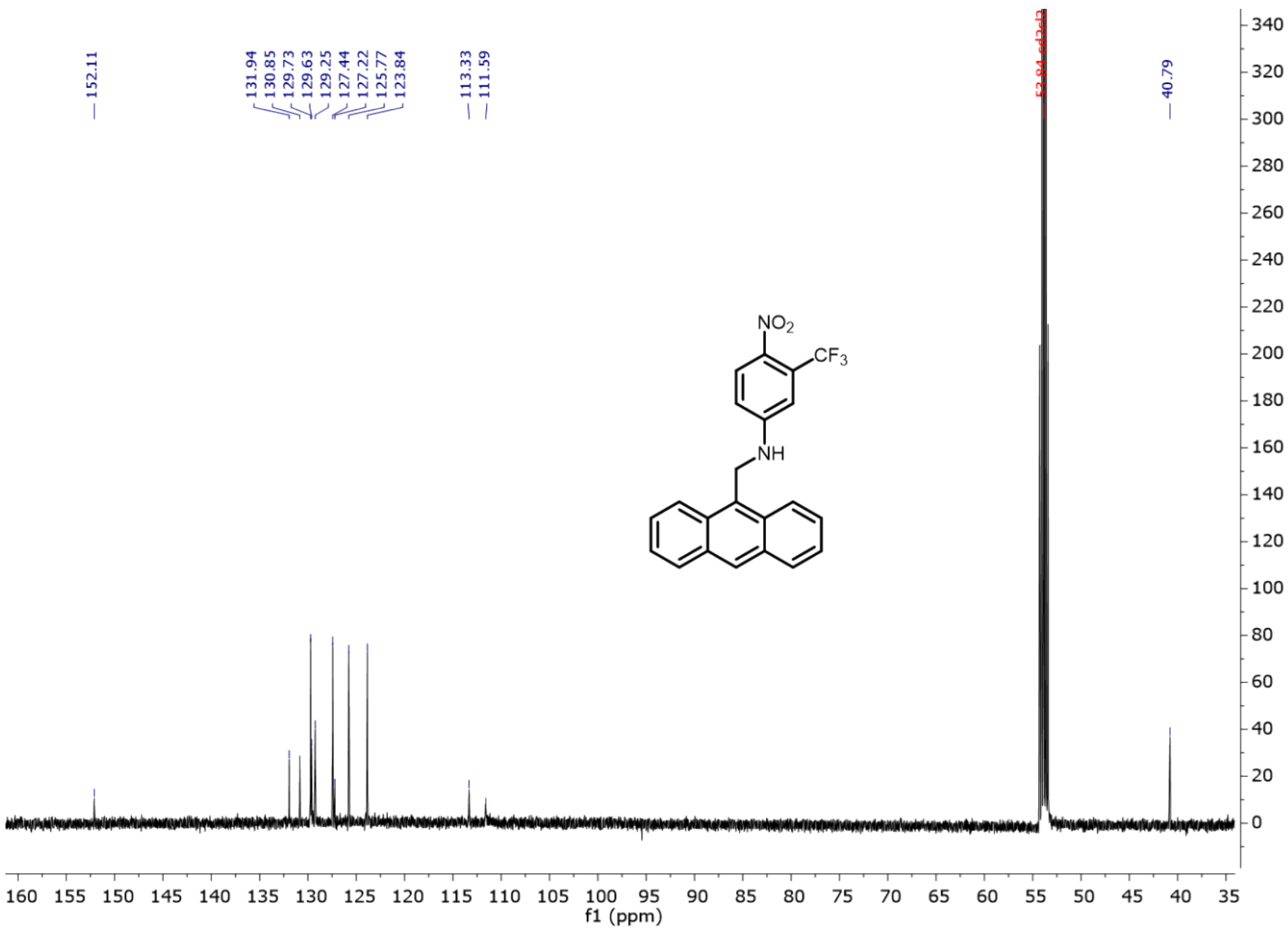

Fig. S2 ^{13}C-NMR spectrum of **An-NOP2** in CD_2Cl_2.

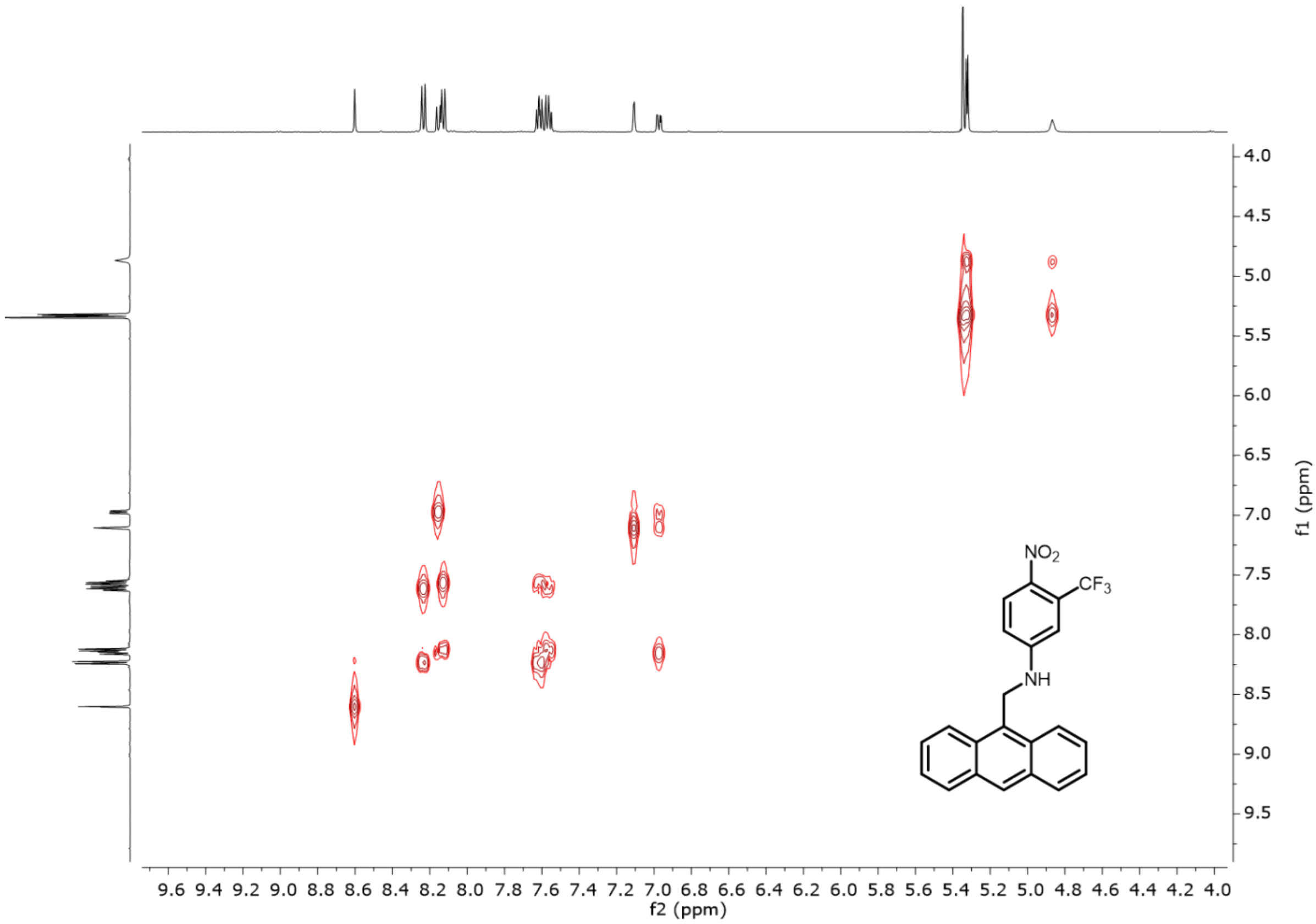

Fig. S3 gCOSY of **An-NOP2** in CD_2Cl_2.

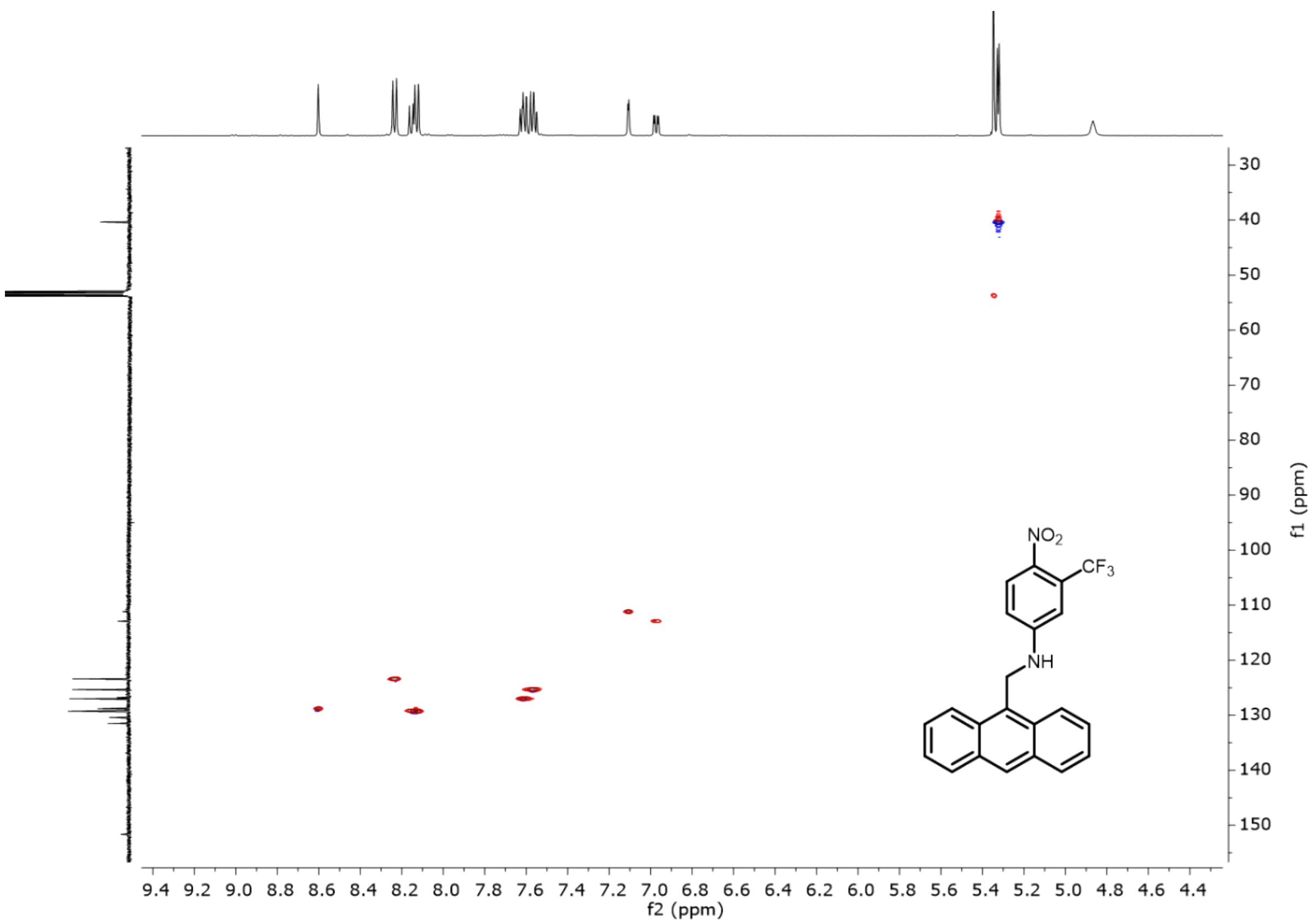

Fig. S4 HSQC spectrum of **An-NOP2** in CD_2Cl_2.

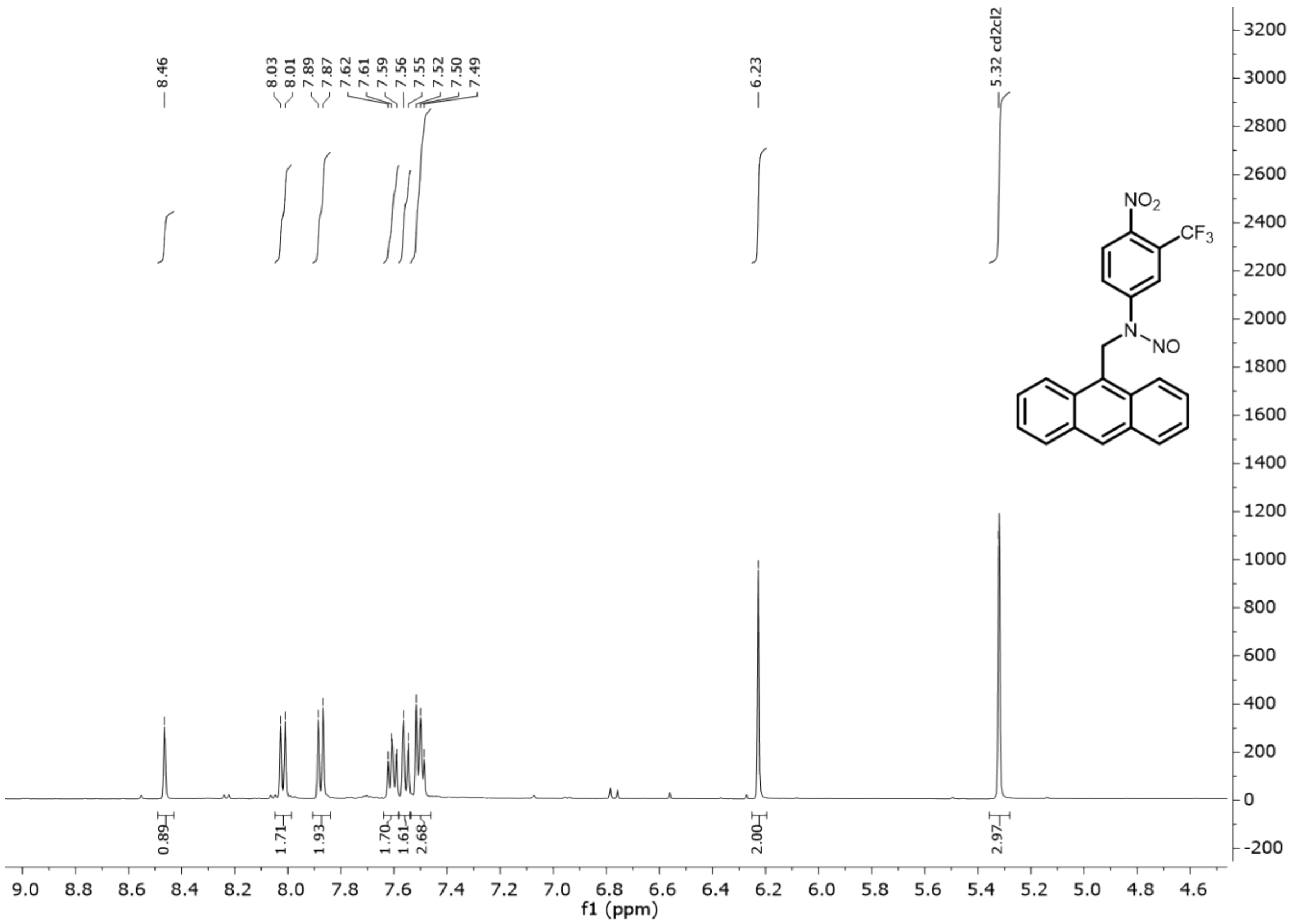

Fig. S5 ^{1}H-NMR spectrum of **An-NOP1** in CD_2Cl_2.

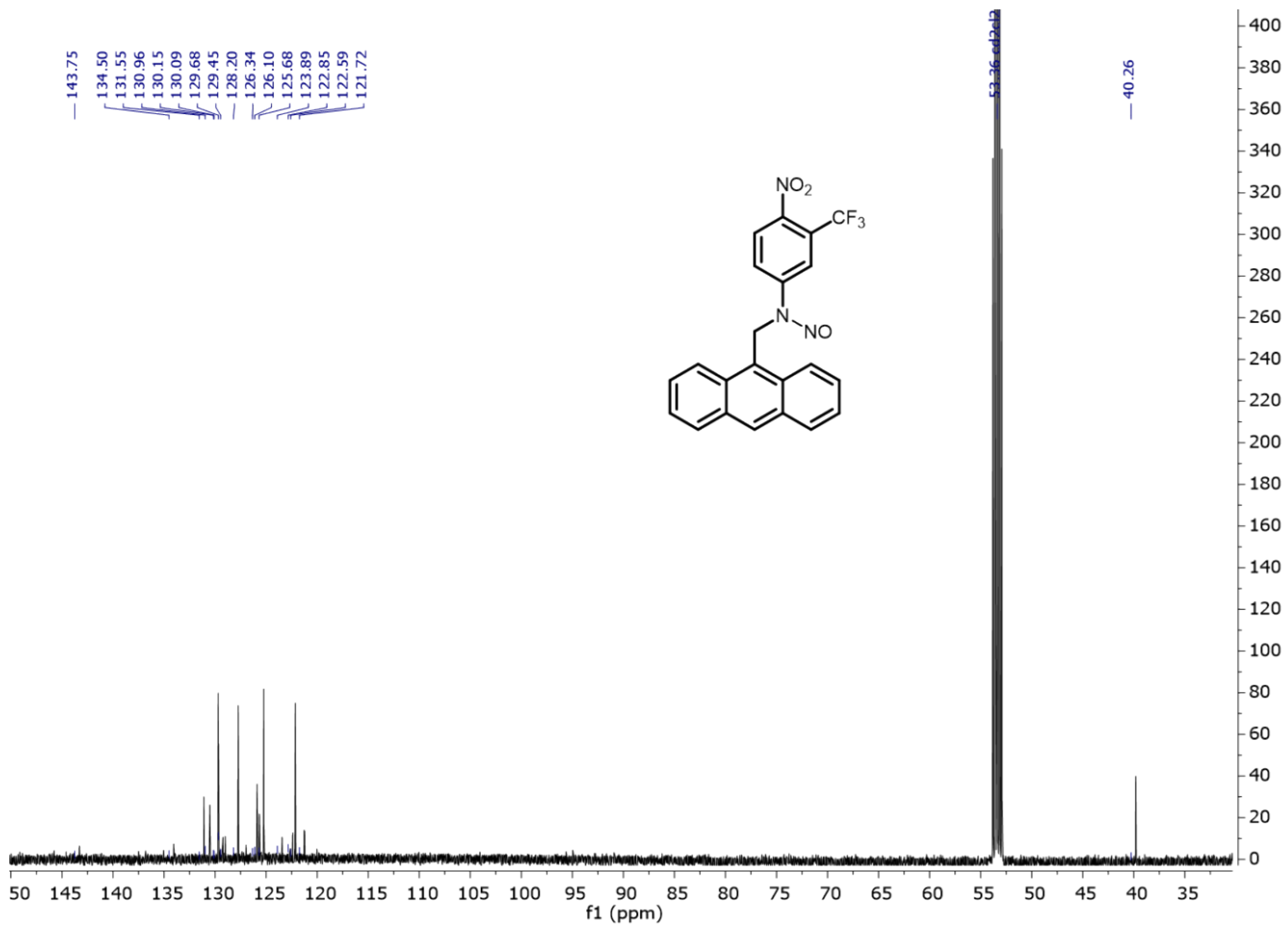

Fig.e S6 ^{13}C-NMR spectrum of **An-NOP1** in CD_2Cl_2.

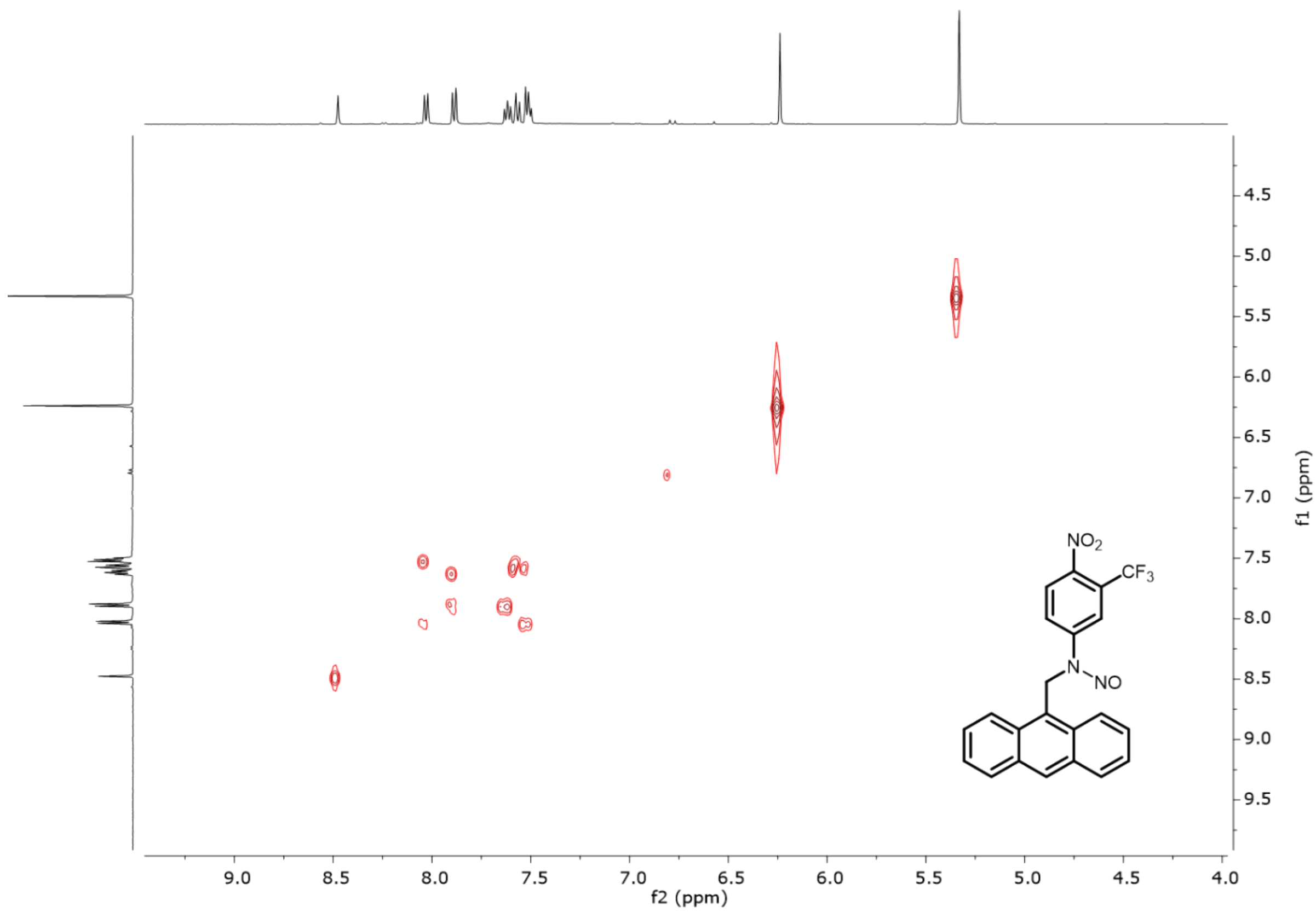

Fig. S7 gCOSY of **An-NOP1** in CD_2Cl_2.

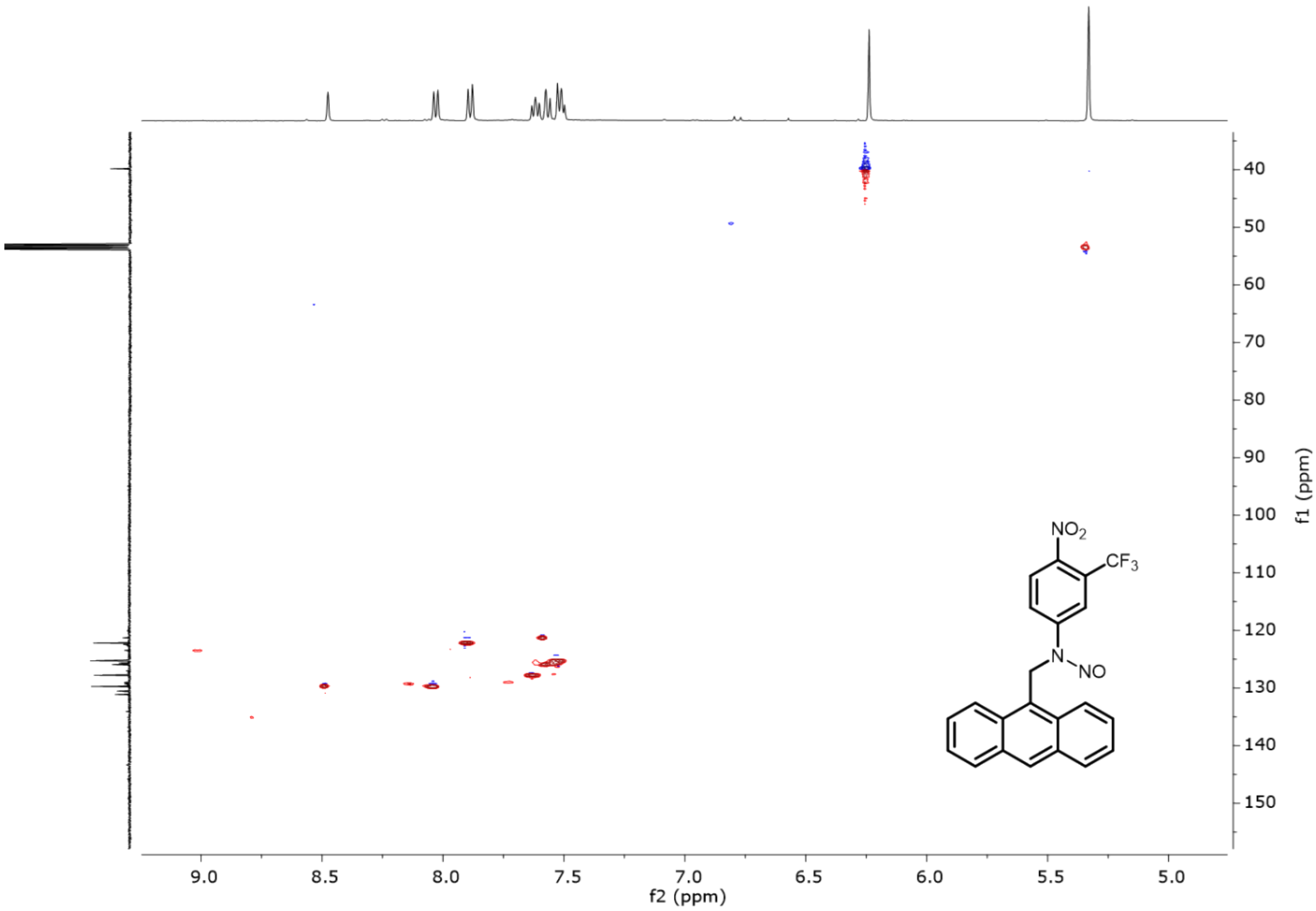

Fig. S8 HSQC spectrum of **An-NOP1** in CD_2Cl_2.

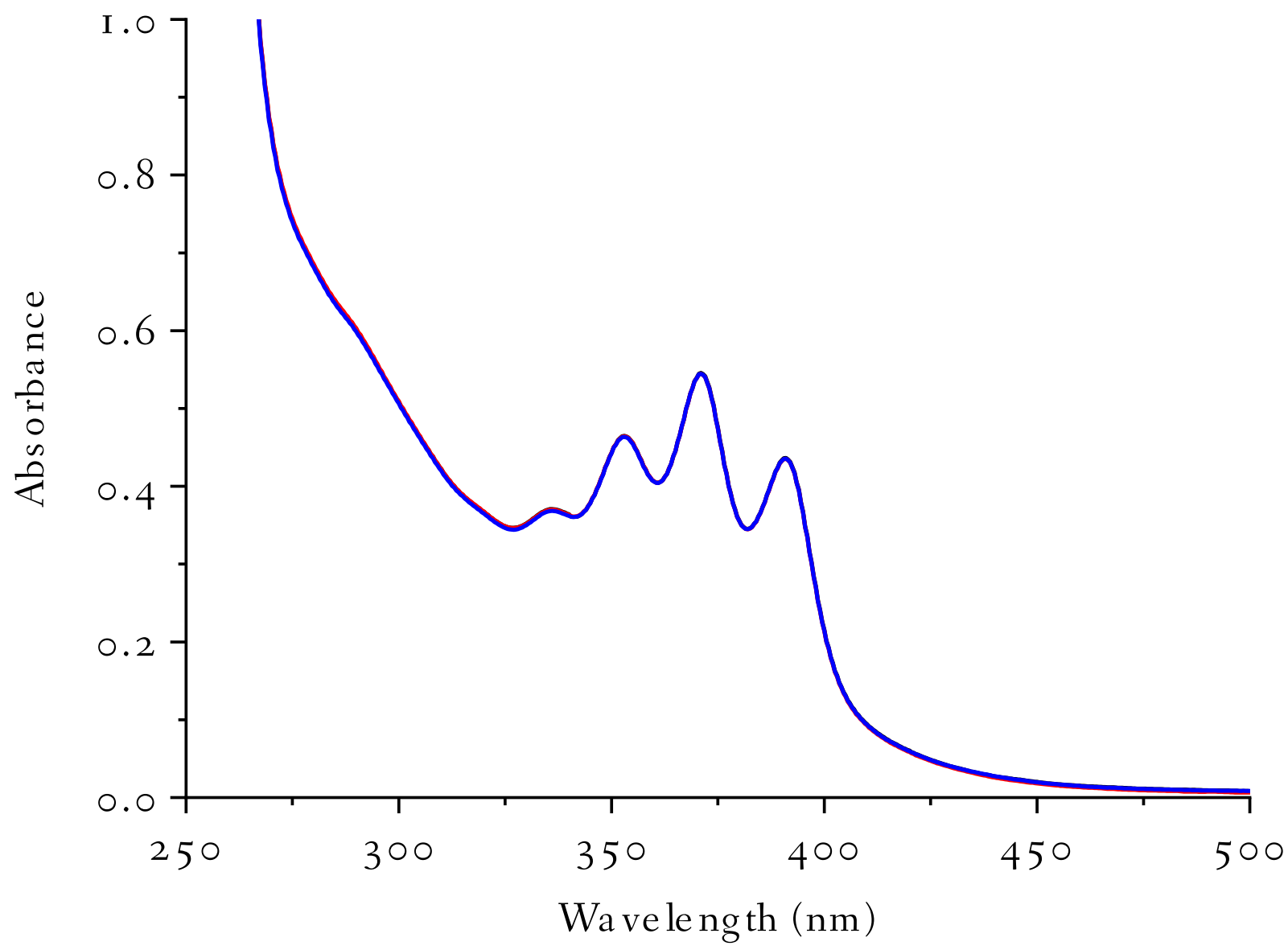

Fig. S9 Absorption spectra of a 50 μM solution of **An-NOP1** (CH_3OH:H_2O 75:25 (v/v) recorded before and after storing one for one week in the dark at room temperature.

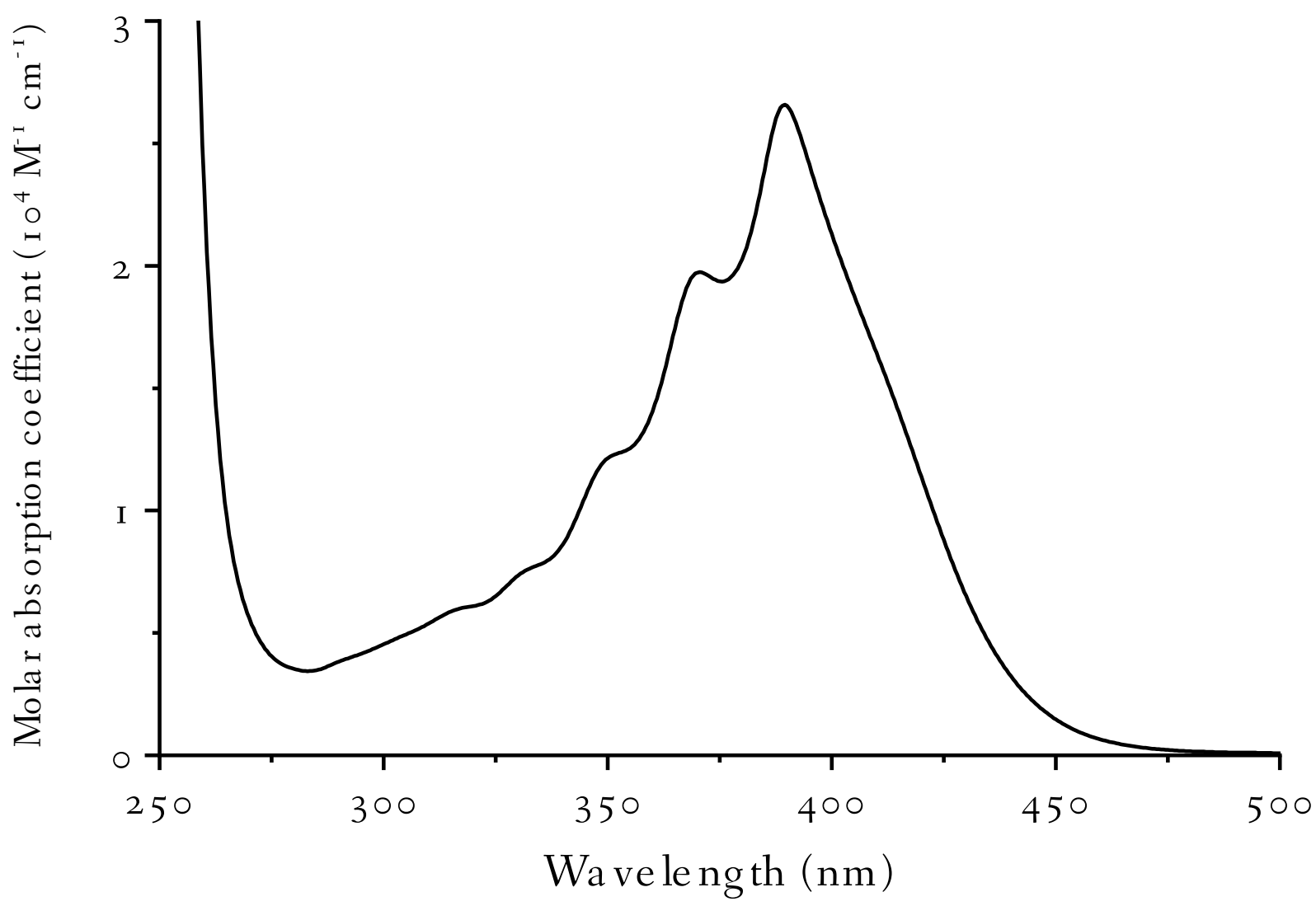

Fig. S10 Absorption spectra of a solution **An-NOP2**. CH_3OH:H_2O 75:25 (v/v); T = 25 °C.

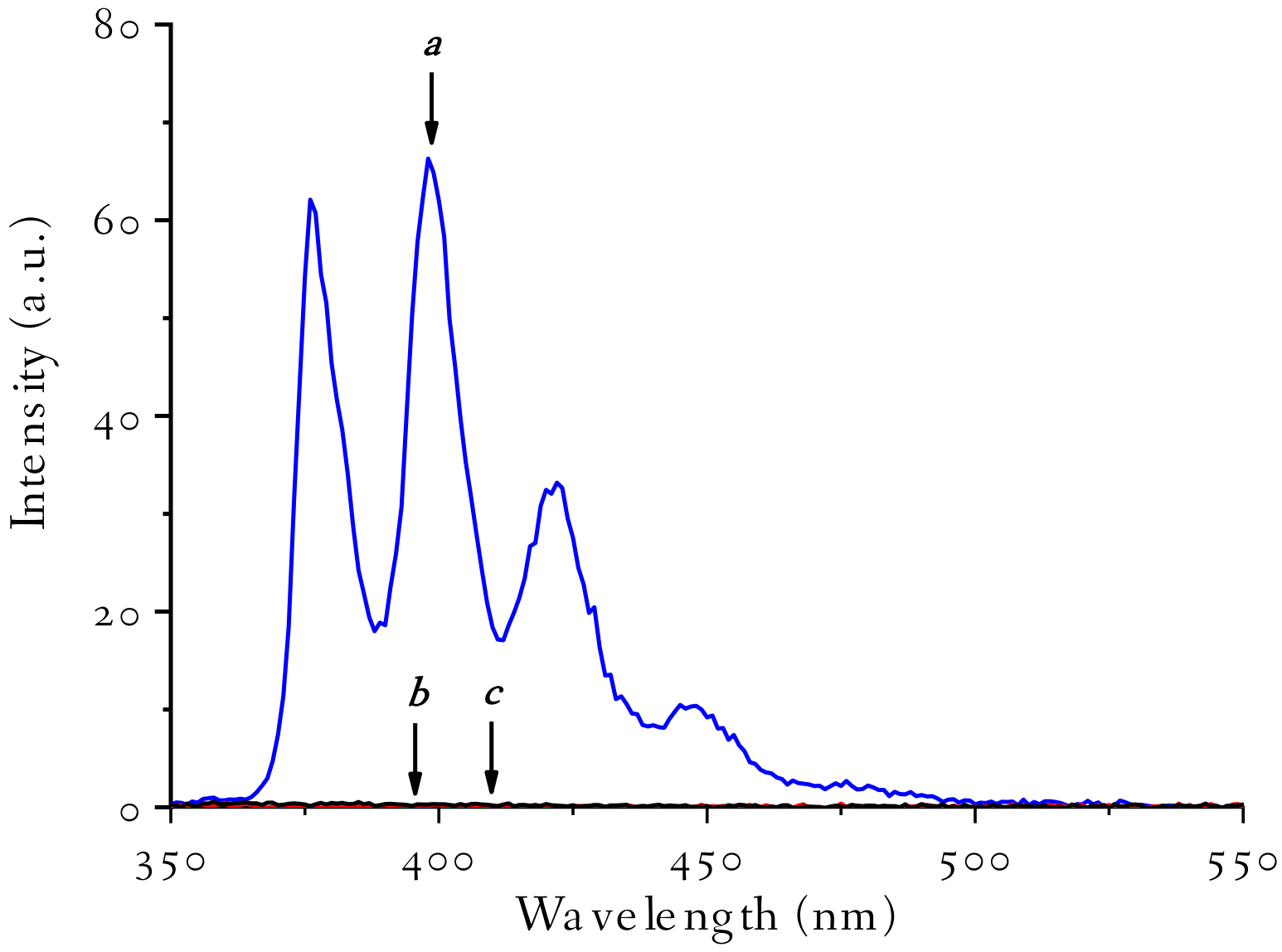

Fig. S11 Fluorescence emission spectra (λ$_{exc}$ = 325 nm) of optically matched solutions of **An** (*a*), **An-NOP1** (*b*) and **An-NOP2** (*c*). $CH_3OH:H_2O$ 75:25 (v/v); T = 25 °C. Note: Note: the fluorescence spectra of **An-NOP1** and **An-NOP2** cannot be distinguished from the wavelength axis.

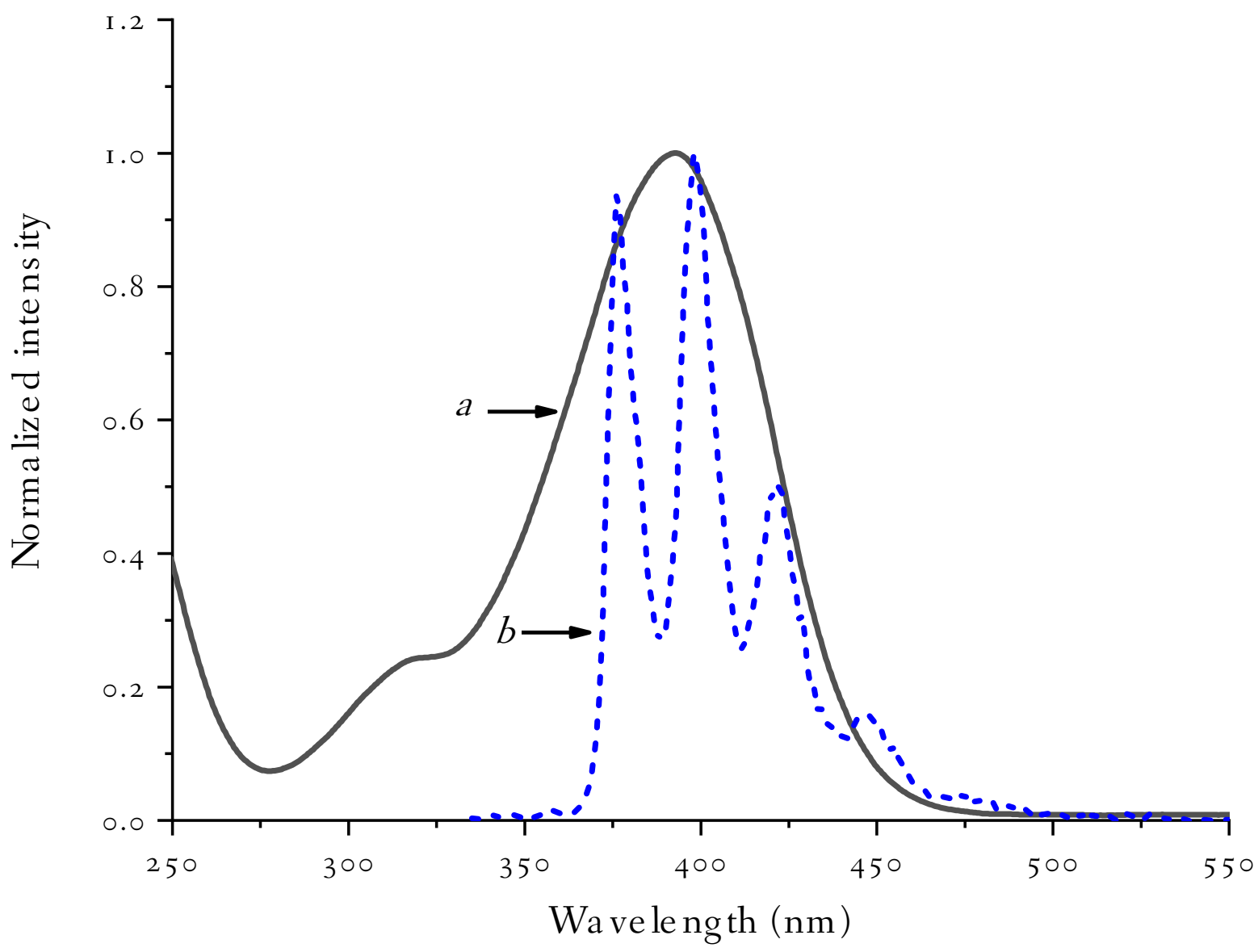

Fig. S12 Absorption spectrum of NOP 1 (*a*) and fluorescence emission spectrum of **An** (*b*), used as model compounds for the dyad **An-NOP2**. $CH_3OH:H_2O$ 75:25 (v/v); T = 25 °C.
